# Blood management in fast-track orthopedic surgery: an evidence-based narrative review

**DOI:** 10.1186/s13018-019-1296-5

**Published:** 2019-08-20

**Authors:** Federico Pennestrì, Nicola Maffulli, Paolo Sirtori, Paolo Perazzo, Francesco Negrini, Giuseppe Banfi, Giuseppe M Peretti

**Affiliations:** 1 IRCCS Orthopedic Institute Galeazzi, Scientific Direction, Milan, Italy; 2 Department of Musculoskeletal Disorders, School of Medicine and Surgery, University of Salerno, Fisciano, Italy; 3 San Giovanni di Dio e Ruggi D’Aragona Hospital “Clinica Orthopedica” Department, Hospital of Salerno, Salerno, Italy; 4 Queen Mary University of London, Barts and the London School of Medicine and Dentistry, Centre for Sports and Exercise Medicine, London, England; 5 Vita-Salute San Raffaele University, Scientific Direction, Milan, Italy; 6 University of Milan, Department of Biomedical Sciences for Health, Milan, Italy

**Keywords:** Blood management, Fast-track surgery, Orthopedics, Joint replacement, Health care value, Ethics

## Abstract

**Background and purpose:**

Innovations able to maintain patient safety while reducing the amount of transfusion add value to orthopedic procedures. Opportunities for improvement arise especially in elective procedures, as long as room for planning is available. Although many strategies have been proposed, there is no consensus about the most successful combination. The purpose of this investigation is to identify information to support blood management strategies in fast-track total joint arthroplasty (TJA) pathway, to (i) support clinical decision making according to current evidence and best practices, and (ii) identify critical issues which need further research.

**Methods and materials:**

We identified conventional blood management strategies in elective orthopedic procedures. We performed an electronic search about blood management strategies in fast-track TJA. We designed tables to match every step of the former with the latter. We submitted the findings to clinicians who operate using fast-track surgery protocols in TJA at our research hospital.

**Results:**

Preoperative anemia detection and treatment, blood anticoagulants/aggregants consumption, transfusion trigger, anesthetic technique, local infiltration analgesia, drainage clamping and removals, and postoperative multimodal thromboprophylaxis are the factors which can add best value to a fast-track pathway, since they provide significant room for planning and prediction.

**Conclusion:**

The difference between conventional and fast-track pathways does not lie in the contents of blood management, which are related to surgeons/surgeries, materials used and patients, but in the way these contents are integrated into each other, since elective orthopedic procedures offer significant room for planning. Further studies are needed to identify optimal regimens.

## Introduction

The demand for total joint arthroplasty (TJA) is going to increase between 174 and 673% by 2030 [1, 2]. In the USA, orthopedic procedures account for up to 10% of all packed-red cell transfusions in a year, 39% of which are used in TJA [3]. Blood loss can be extensive, and allogenic blood transfusion (ABT) is commonly performed. ABTs are associated with risks and complications [3–7]: to try and prevent them, it is necessary to invest in increasingly expensive safety procedures [8]. Moreover, the potential side effects of ABTs also impact social costs [9], accounting for the high economic cost of ABTs.

Innovations able to maintain patient safety while reducing the amount of ABTs therefore add value to orthopedic surgery procedures, where value is defined as outcomes achieved per money spent [10]. Opportunities for improvement arise especially in elective procedures, where room for planning is available. Indeed, experience of blood management in 2511 elective procedures reduced the amount of ABTs without increasing the number of recoveries and readmissions, generating an annual saving of $ 480,000 [11].

Blood management is a pillar of innovative health care models such as bundled payments [12] and fast-track pathways [13–15], as perioperative coordinated care is associated to a better and quicker recovery [15]. Accurate blood management can contribute to home discharge of 85-year-old patients 3 days after surgery[16], as well as to safely perform procedures in outpatient settings [17].

Although many strategies and algorithms have been proposed to reduce ABTs, there is no consensus about the most successful combination, given the different opinions over the same procedure, and the need to adapt the procedures to the characteristics of individual patients [18–20].

The purpose of this investigation is to identify information to support blood management strategies in fast-track TJA pathway, to (i) support clinical decision making according to current evidence and best practices, and (ii) identify critical issues which need further research.

Reviews on blood management in orthopedics surgery [3], elective orthopedic surgery [8], total hip and knee arthroplasty [18, 21], and lower limb joint arthroplasty in a bundled payment [11] have been published. However, no review, to our knowledge, focuses on blood management in fast-track orthopedic pathways, except for general European Guidelines on perioperative venous thromboembolism prophylaxis [22].

Such a review should be helpful in two ways: first, by elaborating a synthesis on what has been published on the topic; second, by pointing out which parts of the perioperative process offer room for improvement.

## Materials and methods

We proceeded in four steps.

First, we identified conventional blood management strategies in elective orthopedic procedures, to achieve a general road map to support the research on fast-track innovations. Six articles addressed the perioperative approach to the topic [3, 8, 9, 18, 21, 23]. To provide a clear roadmap and adhering to previous recommendations [24–26], the strategies were divided in preoperative, intraoperative, and postoperative [27].

Second, we searched PubMed, Embase, Ebsco host, and Cochrane reviews for blood management strategies related to fast-track total joint arthroplasty, using the following keywords: fast(-)track surgery, orthop(a)edics, total joint arthroplasty, blood, blood management. After removing duplicates and studies not related to TJA procedures, 16 articles were eligible. These were read in full text to ensure their consistency with a fast-track TJA pathway.

Third, we designed tables to match every step of the perioperative process with information from the fast-track literature. In each table, information reported in the central column is therefore coming from conventional procedures. When it was not possible to fill the relevant column of the tables with information on fast-track joint arthroplasty arising from the second step, we performed a further search on the topic using PubMed, Embase, Ebsco host and Cochrane reviews databases, bibliographies, and articles suggested by colleagues. These results—which are useful both for clinical and further research—are highlighted in the table with a *.

Fourth, we submitted the information reported in the right column to the opinion of clinicians who operate using fast-track surgery protocols for TJA at our hospital, to (i) check whether the information was sound, (ii) offer them opportunities for improvement, and (iii) identify practices which they already used, as such practices may be not reported in the published literature.

We acknowledge that steps three and four prevent the research from being entirely reproducible, which represents the main limitation of our study, but we believe this disadvantage to be offset by the advantage of providing information which can be missed with conventional research. We therefore opted for a narrative rather than a systematic review. Accordingly, we have adopted four of the six Cochrane recommendations for a systematic review27 (Table 1).

**Table 1:** Cochrane guidelines for systematic review

1	Formulation of a clear question	Which procedures support the implementation of blood management in fast-track total joint arthroplasty?
2	Exhaustive and reproducible research of all relevant information (published and unpublished studies) concerning the question	Partly reproducible, non-exhaustive.
3	Systematic selection of eligible studies on the basis of pre-defined inclusion criteria	All papers had to be related to fast-track pathways or accelerated procedures, and had to be written in English at least in the abstract. Detailed information is reported in the PRISMA diagram before the end of introduction.
4	Analysis of the methodological quality of included studies	No.
5	Quantitative or qualitative synthesis of the information according to the nature, complexity of the question and availability of data	To facilitate reading, qualitative and quantitative information is synthesized and summarized in tables. Information about fast-track procedures is matched with every single step of the general orthopedic blood management strategy.
6	Discussion of the reasons for agreement and inconsistency between the results of the different studies	Yes.

All searches were completed by January 21st, 2019.

## Results

### Preoperative blood management

Preoperatively, patients are thoroughly assessed, to decide whether to include them in an accelerated pathway, to plan clinical contingencies, and to educate the patients to take active part in the process. All are key steps in a successful fast-track design (Table 2).

**Table 2:** Preoperative blood management

Content	Conventional studies, guidelines, and recommendations	Fast-track setting
Anemia and iron deficiency treatment Since preoperative and postoperative anemia are generally related, we refer to the latter in the present section.	Anemia standards and detection	
	Patients undergoing TKA should meet standard criteria regarding the minimum preoperative Hb [28–30]. Otherwise, surgery should be postponed [29]. Recent guidelines recommend preoperative correction of anemia and iron deficiency in all patients with a Hb < 13 g/dL [30]. Preoperative assessment of anemic patients should be performed between 30 and 60 days before the procedure, in order to investigate the cause and plan the ideal treatment [24–26, 29, 32–34].	Pre- and postoperative Hb levels (together with the subsequent need for transfusions) play a role on fast-track TJA postoperative outcomes, Length of Stay (LOS) and patient satisfaction [15, 35]. Preoperative anemia is frequently associated with prolonged LOS, 90-day re-admission and blood transfusions, preventing fast-track TJA to express full value both in THA and TKA procedures [31, 36] (*). 549 fast-track TJA procedures on patients aged ≥ 85 years revealed blood-related issues such as postoperative anemia, blood transfusions, and mobilization to be the most relevant medical cause of more than 4 days LOS (27.3%), where preoperative anemia had no significant impact. Preoperative anemia was instead one of the main medical causes of readmissions within 90 days, together with suspected but disproved thromboembolic events. Authors concluded that fast-track TJA aiming at 3 days median LOS and discharge to home is feasible in most patients aged ≥ 85 years, provided attention to pre- and post-operative anemia [16]. With regard to THA, authors show a weak but significant correlation between post-operative Hb and early functional recovery (6-min walk test) in 65 year-old patients, while they did not find any influence between the former and other functional criteria nor quality of life [37].
	Iron deficiency treatment	
	Iron-deficiency anemia is the main cause of low Hb [38, 39]. This is why preoperative iron supplementation is highly suggested in orthopedic procedures, alone or together with intraoperative tranexamic acid (TXA) [40, 41]. (*) Studies report intravenous (IV) iron therapy to be safe and dominate on oral, in particular with patients with malabsorption such as coeliac disease [32, 42, 43]. When using oral supplements for iron storage, a daily dose of 100 mg elemental iron is recommended for 2 to 6 weeks before surgery. When using IV iron supplementation, requirements can be estimated using the Ganzoni equation [44].	Studies on 882 unselected fast-track THA/TKA patients confirmed anemia to be prevalent in elderly patients and to be associated with increased transfusion risk and postoperative morbidity. Iron deficiency is confirmed to be the most common and reversible cause [45]. Fast-track THA non-anemic patients with iron deficiency were supplemented with oral highly absorbable Sideral® Forte (at least) 4 weeks before surgery. They had a smaller decline in post-operative Hb, shorter hospital stay and reduced blood transfusions, generating a saving of 1763.25 € per patient. Therefore, preoperative sucrosomial iron® is a cost-effective solution for fast-track THA surgery [46]. (*)
Blood thinners (antiplatelet, anticoagulant, antithrombotic agents)	These drugs have a key role in preventing cardiac and vascular events [47]. To normalize bleeding, they are usually stopped a few days before surgery [8]... ... But patients at high cardiovascular risk should not stop aspirin in the perioperative period [48]. Given that cardiovascular disease is common in patients planning to undergo to TKA, both the continuation and the discontinuation of antiplatelet therapy can be associated with major risks, depending on patient characteristics, severity of the procedure and estimated time of immobilization [8, 49, 50]. The management of these medications in the perioperative setting should be adapted to the single patient according to cardiologist, orthopedic surgeon, and anesthesiologist [18].	Preoperative use of anticoagulant agents needs important evaluations in fast-track TJA too [51, 52]. (*) A study on the incidence of stroke within 30-days after 24.682 fast-track TJA found preoperative use of anticoagulant treatment to be the most important risk factor, together with age ≥ 85. Anemia was also included, but not significant. It is therefore important to check for the use of preoperative anticoagulants, and anemia, to avoid cardiovascular perioperative events in elderly patients [53]. (*)
Transfusion protocol agreement Although transfusion is a postoperative intervention, establishing the trigger must be done before surgery.	Reasons to variate transfusion practice in orthopedic surgery are not well understood. In case of elective surgery, the need for allogenic transfusion was said to predictable in 97.4% of the cases, according to (i) preoperative anemia, (ii) perioperative blood loss, (iii) transfusion trigger [54]. A survey on clinicians and hospitals in the UK showed transfusion triggers after TJA to variate between 6 to 11 g/dL, calling for shared evidence-based guidelines to improve practice and avoid waste [55]. This is consistent with results from a previous international survey, according to which transfusion triggers vary significantly among different clinicians, hospitals and countries [56]. Systematic review on transfusion drivers in orthopedic surgery shows low Hb and old age to be the main predictors for the need of transfusion, followed by surgical complexity, low body weight, additional comorbidities (rheumatoid arthritis, history of anemia, diabetes, cardiovascular disease, renal failure, or metastasis), and female sex [57]. Studies on restrictive triggers in major orthopedic patients show transfusion rate and deep wound infections to be respectively reduced from 34 to 17%, and from 2.6 to 1.5% [58]. This is confirmed by a prospective study on unilateral knee arthroplasty (UKA), which adopted a postoperative trigger < 8.5 g/dL (or greater in case of a symptomatic patient): transfusion rates were reduced from 31 to 11.9%, blood waste from 60 to 1%, with no adverse outcomes [59]. Current evidence confirms that a restrictive trigger (Hb < 8 g/dL) is safe and cheaper^3^ ... which is supported by the National Institute for Health and Clinical Excellence even more restrictive recommendations [60].	A study on fast-track TJA predictors of LOS and patient satisfaction found: The need for blood transfusion to be the main predictor of a longer stay (> 3 days) Blood transfusion to occur in 22% and 12% of THA and TKA respectively, although it does not compromise a high satisfaction rank (9.4 and 9.3 up to 10) Transfusions were administered when postoperative hematocrit level (at the first day) was 25% less than preoperative. Transfusions were associated with age, lack of mobility during the first day after surgery, co-morbidities, low Hb and increased ASA score [15]. The need for blood transfusion is a predictor that produces significant value in accelerated pathways such as fast-track surgery, since they provide caregivers with useful information to plan for treatments and beds. Waiting for blood transfusion is indeed one of the main reasons to delay discharge [61, 62]. (*)
Erythropoietin (EPO)	EPO reduces post-operative transfusions [63] both in THA and TKA [64–67], even in rheumatoid arthritis patients [67], but it is associated with adverse events such as deep venous thrombosis (DVT), pulmonary embolism (PE), fever, hypokalemia, urinary tract infection, nausea, hypoxia, and vomiting in up to 5% of the population [68–70]. Moreover, it is unclear whether benefits are not offset by costs [71]. Therefore, EPO is suggested in exceptional conditions such as patients with strong anemia who (i) cannot receive blood because of red cell antibodies, (ii) refuse donation because of religious beliefs [18, 60, 72].	The use of TXA has almost eliminated the need for other blood conservation strategies [73, 74], and is therefore a valuable innovation to be applied under a fast-track pathway [75].
Preoperative autologous blood donation (PAD)	Major concerns regarding PAD are related to handling errors, blood infection, and poor cost-effectiveness [76–79]. The overall benefits of PAD in primary joint arthroplasty can outweigh the harms in alloimmune and rare blood types patients [8, 80].	The use of TXA has almost eliminated the need for other blood conservation strategies [73, 74], included PAD, which makes it a valuable innovation under a fast-track pathway[75].

### Intraoperative blood management

Intraoperative management plays the most important role in safety, blood loss minimization, and early recovery (Table 3) [18].

**Table 3:** Intraoperative blood management

Content	Conventional studies, guide lines, and recommendations	Fast-track setting
Minimally invasive surgery (with or without navigation)	MIS techniques are believed to reduce blood loss in TJA procedures, included the so-called tissue sparing surgery [81–85]. Blood loss after minimally invasive TKA was compared between using or not imageless navigation, together with intraoperative tourniquet but no postoperative drainage. Blood loss was not significantly affected by the use of imageless navigation, following which time of surgery was a bit longer but Hb reduction and amount of blood were similar [86].	Blood loss reduction is included in the benefits of MIS TJA surgery. However, a survey on fast-track TJA procedures concludes that MIS benefits on blood loss are still unclear [87], and more studies should be conducted to assess whether its use in fast-track THA affects overall patient satisfaction [88] (*).
Tourniquet	Although the majority of orthopedic surgeons still widely use it, its role remains controversial. If tourniquet reduces intraoperative blood loss, this gain can be offset by the amount of blood lost after its release [89]. A meta-analysis on 30 RCTs seems to confirm its lack of effectiveness in TKA for better clinical outcomes, less complications and better early post-operative ROM achieved *without* a tourniquet [90].	The benefits of tourniquet in TKA are also questioned in fast-track surgery. An RCT found knee-extension 48 h after surgery to be reduced in 90% patients regardless to its use. Moreover, tourniquet reduced bleeding during surgery, but had no benefits on postoperative Hb levels, pain, nausea, OS, or periarticular swelling. Finally, using or not a tourniquet had no difference in early postoperative outcomes after surgery [91]. A study on 151 fast-track TKA verified the effectiveness of a tourniquet on post-operative bleeding and rehabilitation together with suction drainage application. Suction drain was associated to lower Hb levels, higher transfusion rate, higher pain and slower functional recovery, while short-term tourniquet did not influence postoperative bleeding and rehabilitation program [92]. (*) However, a protocol for another RCT aims to verify the effectiveness of a tourniquet on patient’s recovery after fast-track TKA, in association to the anaesthetic regimen. Primary outcome is cumulative intravenous oxycodone consumption by patient-controlled analgesia during the first 24 postoperative hours. Secondary outcomes include postoperative nausea and vomiting, the length of hospital stay, the duration of the surgery, blood loss, demand for surgical unit resources, complications, readmissions, postoperative knee function, range of motion, health-related quality of life, prolonged pain, and mortality [93].
Anesthesia	In order to provide guidelines for fast-track TKA, it was conducted a survey on anaesthetic techniques [94]. With regard to blood management, it was found that blood loss is not affected by administering regional (RA) or general anesthesia (GA), while RA is associated with other outcomes such as reduced post-operative pain, length of stay and better rehabilitation [95]. RA is associated with lower thromboembolic complications, even if—after performing a subgroup analysis—anticoagulants were the precaution who made the difference [96]. RA is suggested for TKA patients with comorbidities [97], while there is no evidence enough about their benefits on cardiovascular morbidity, DVT, and PE in association with pharmacological thromboprophylaxis [95]. In case of THK, the same authors did not find evidence enough to compare the benefits of GA or RA on blood loss [98], while others found RA to be better after adjusting for patients’ specific comorbidities and/or when combined with accurate transfusion prevention [99, 100].	With regard to intraoperative blood loss, no difference was found in performing GA (through propofol and remifentanil) or spinal anesthesia (SA) (through intrathecal bupivacaine) However, GA is confirmed to dominate on SA on relevant outcomes for a fast-track pathway, such as early mobilization, less opioid consumption, and reduced pain scores 6h after surgery [101].
Hypotensive epidural anesthesia (HEA)	HEA was developed to combine the advantages of epidural anesthesia (airway problems, reduced rate of DVT) with the benefits of induced hypotension [102]. Although HEA’s use seems to be safe and effective, it’s not a first line method in TKA, while it is more spread in THA [103–105].	There seems to be no reason to choose or not for HEA according to the pathway. Searching the literature for #hipothensive epidural anaeshtesia, #hea, #arthroplasty and #fast(-)track gave no results.
Antifibrinolytic agents	The most common antifibrinolytic agents in use are Tranexamic Acid (TXA) and ε-aminocaproic acid (EACA) [106–108]. TXA is more cost-effective than EACA on reducing perioperative bleeding and transfusions [109]. Apoproptine is more effective at decreasing blood loss, but increase the risk of cardiovascular complications. Therefore, it has been removed from the market [110–112]. TXA seems therefore to be the best solution. Indeed, meta-analysis show the use of TXA in TKA to be and effective and safe solution in reducing blood loss [113]. A RCT proved TXA to dominate on post-operative cell salvage both in primary THA and TKA [114], which is confirmed by several studies, even if the ideal regime remains controversial, and variates according to topical (intra-articular), general (intra-venous), and amount of administration [73, 74, 115–120]. Multiple intravenous boluses injections (pre-, intra-, postoperatively) proved to dominate on a single intravenous dose [121], as well as a bolus of tranexamic acid followed by infusion was found to be more useful than a single dose in decreasing perioperative blood loss in patients undergoing hip surgeries: it reduced allogenic blood transfusions without increasing risk of thromboembolic events [122]. Intra-articular administration is a safe alternative for TKA patients at risk for intravenous administration [123]: moreover, one intra-articular administration is as effective as three doses regimen in preventing blood loss with no difference in thromboembolic complications [124, 125]. According to another study, mixing IV and IA administration is better than administering them alone [126, 127]. RCTs show the high effectiveness of TXA, both with tourniquet and without [117, 118]. Finally, there is no contraindication for its use in patients with a history of venous thromboembolism [128]. Tranexamic acid has been recognized as a valuable innovation under a bundled payment model [11] (*).	Fast-track TJA was soon declared to produce no more complications than conventional procedures, including thromboembolic episodes [129]. TXA is shown to reduce perioperative blood loss (and the following need for allogenic blood transfusion) also in accelerated recoveries and clinical pathways, without increasing the rate of thromboembolism [14, 92, 130, 131]. (*) Combined intra-articular (IA) and intravenous TXA reduced blood loss in 60 fast-track TKA patients [132]. Adding low-dose epinephrine to TXA on 100 fast-track THA patients did not reduce blood loss during surgery, but reduced it of 180 mL within 24 h [133]. (*) Tranexamic acid is recognized as a valuable innovation under a fast-track THA pathway [75]. Its effectiveness in conventional TKA had also been largely demonstrated [114, 134], supporting it to be a valuable innovation in fast-track TKA too.
Topical fibrin sealants (TFS)	A meta-analysis on TKA suggests the administration of intravenous TXA to dominate on TFS [135], whose effectiveness and cost-effectiveness are debatable [136, 137].	The eventual use of TFS in revision surgery makes them irrelevant for fast-track pathways, which are dedicated to primary intervention.
(Intraoperative) cell salvage	A meta-analysis on 43 trials show perioperative (intra- and post-) cell salvage to reduce blood loss in both THA and TKA [138]. However, the study points out how more recent trials show blood salvage benefit to be overwhelmed by more effective innovations [139–141].	The use of TXA has almost eliminated the need for other blood conservation strategies [73, 74], included intraoperative blood salvation, and is therefore a valuable innovation to be applied under a fast-track pathway [75]. Intraoperative cell salvage is still useful to those patients who cannot be administered TXA.
Peri/intra-articular injections	Topical hemostatic vasoconstriction	
	Epinephrine is the agent of choice for its topical hemostatic vasoconstriction [142]. Injections of epinephrine together with bupivacaine just before wound closure reduced 32% of drain output, but showed no significant reduction in transfusion rate [143]. Moreover, a recent study denies the effective hemostatic role of intra-articular epinephrine in TKA [144]. However, the combined administration of low-dose epinephrine and tranexamic acid reduced perioperative blood loss and inflammatory response compared with tranexamic acid alone, with no apparent increase in thromboembolic and other complications [145].	A synthesis on fast-track TJA clinical and organizational aspects questioned the value of adding epinephrine during anesthesia [129]. Adding epinephrine to TXA on 100 fast-track THA patients did not reduce intraoperative blood loss, but reduced it of 180 mL within 24 h [133] (*), confirming perioperative blood loss to be reduced by the combination between intravenous low-dose epinephrine and tranexamic acid.
	Local infiltration analgesia (LIA)	
	A review over 11 RCTs shows LIA to be a safe and efficient technique for TJA [146]. A systematic review over 27 RCTs confirmed LIA to provide effective analgesia in TKA, either combined or not with multimodal systemic analgesia; in contrast, LIA provided limited additional benefit in THA when combined with a multimodal regimen [147]. The introduction of intraoperative LIA is consistent with blood management for its combination with TXA proved significant benefits on postoperative Hb levels, reduced LOS, no increase in transfusion and, therefore, better cost-effectiveness (when compared to perioperative autologous blood salvage and preoperative EPO) [148].	Postoperative pain treatment is a fundamental step in achieving early rehabilitation and reduced hospital LOS, therefore the use of LIA is ideal in fast-track pathways [147] (*), even more in combination with TXA, whose benefits were discussed in the dedicated section.
Bipolar and monopolar sealants	Bipolar sealant is a novel approach in TKA, but it provides no significant difference in postoperative drain output, Hb level and transfusion requirement when compared to monopolar sealant [149–155]. As long as the cost-effectiveness of bipolar sealants remains controversial, there is no reason to adopt them in routine care [156].	A synthesis on fast-track TJA clinical and organizational aspects included bipolar sealants as a means to minimize blood loss and transfusions [129]. However, there seems to be no particular reason to choose or not for bipolar or monopolar sealants according to the pathway. Searching the literature for #bipolar sealant, #monopolar sealant and #fast(-)track gave no results.
Platelet-rich plasma (PRP)	Intra-operative adoption of PRP is shown to reduce TKA post-operative blood loss [157]. PRP is effective in wound healing, but controversial in haemostasis [158, 159].	There seems to be no reason to choose or not for PRP according to the pathway. Searching the literature for #platelet rich plasma, #PRP, #blood and #fast(-)track gave no results.
Bone wax	Bone wax helps to control bleeding from bone surface during surgical procedures [160]. Despite its recent use in TKA to reduce total blood loss while maintaining higher Hb levels, more studies are needed to verify its safety with regard to allergic reactions, inflammation and foreign bodies formation [161, 162].	There seems to be no reason to choose or not for bone wax according to the pathway. Searching the literature for #bone wax, #arthroplasty, #orthopedics and #fast(-)track gave no results.
Sealing femoral tunnel	A meta-analysis over 4 TKA RCTs concludes that the use of extramedullary (EM) guide results in less blood loss—given similar operation time—in comparison with the intramedullary (IM) one [163]. However, IM seems to dominate EM on TKA survivorship [164].	There seems to be no reason to choose or not for sealing femoral tunnel according to the pathway. Searching the literature for #sealing femoral tunnel, # arthroplasty and #fast(-)track gave no results.

Several studies have also investigated the role of intraoperative intermittent pneumatic compression devices (IPCDs) in preventing postoperative venous thromboembolism (VTE), but we report them in the following section because of their association with postoperative pharmacological prophylaxis.

### Postoperative blood management

Fifty percent of the total blood loss in TKA occurs postoperatively [165], especially within the first 4 h (Table 4) [166].

**Table 4:** Postoperative blood management

Content	Conventional studies, guide lines, and recommendations	Fast-track setting
Postoperative anemia	See Table 2.	
Compression	Inelastic compression bandage after TKA seems not to reduce blood loss, but offers a slight improvement in reducing postoperative pain and early functional outcomes [167, 168]. According to other studies, there is no difference in compression method [169–171].	Intermittent pneumatic compression to reduce bleeding in (fast-track) high risk patients is considered later, together with thromboprophylaxis.
Cryotherapy	Systematic review on 13 RCTs proved cryotherapy to be effective in reducing blood loss after TKA selected patients, while its benefits remained controversial after THA [172]. Previous systematic review and meta-analysis over 11 TKA studies show only slight short-lasting benefit of cryotherapy in routine procedures [173].	Cryotherapy has been advocated as a safe and effective strategy to improve fast-track TKA postoperative results, acting on pain, edema, and blood loss. Continuous cold flow device in the acute postoperative setting did not show superiority in comparison with traditional icing regimen. Thus, due to the costs, it should be reserved to selected cases [174].
Limb position	Different knee flexion positions (e.g., hip elevation by 60° combined with 60° knee flexion) have been reported to have promising results with respect to reducing perioperative blood loss [175–177]. Postoperative knee flexion is therefore an easy, inexpensive, and effective method in blood loss reduction [18].	There seems to be no reason to opt for a limb position according to the pathway. Searching the literature for #limb(-)position, #blood and #fast(-)track gave 2 results, none of which consistent with orthopedics.
Postoperative cell savage	PCS effectiveness and cost-effectiveness seem to be maximized in patients with pre-operative Hb 12–15 g/dL, while in patients with pre-operative Hb less than 12 g/dL it should be combined with other techniques [178].	The use of TXA has almost eliminated the need for other blood conservation strategies [73, 74], included postoperative blood salvation, and is therefore a valuable innovation to be applied under a fast-track pathway [75]. Postoperative cell salvage is still useful to those patients who cannot be administered TXA.
Drainage clamping and removal	The introduction of drainage clamping in bilateral total joint arthroplasty proved effective and cost-effective in pioneer studies on TKA and THA [179, 180]. More recent studies on TKA proved its effectiveness to be debatable [181–183], and when effective, proved debatable intervals of administration [184–186]. Today, 3-h interval clamping proved effective in reducing TKA postoperative Hb drop with no increase in thromboembolic episodes and wound complications [187], and proved to be even more effective in combination with TXA, even if there is still need of a major focus on duration and intervals [188, 189]. With regard to THA, 4-h drainage clamping proved potential for routine implementation, for it reduced blood loss and the following need of transfusion with no significant difference on other clinical outcomes [190]. According to a RCT on 224 THA patients, intra-articular soaking of high concentration of TXA with 2-h clamping drainage can reduce the total blood loss and transfusion rates in primary THA without significant increase in postoperative thrombotic complications [191].	A prospective cohort study evaluated the safety and feasibility of early removal of drainage tube in primary fast-track TKA. Wound drainage was removed within 6–12 h after surgery. The procedure could drain the haematocele and reduce the risk of infection, without increasing the sense of pain, inflammatory reaction, limb swelling, and total blood loss. Removal of drainage tube was therefore safe and feasible within 6–12 h after surgery [192]. According to clinical expertise, drains can be avoided in selected cases, when local conditions free of obvious bleeding following re-established pressure ensure the absence of the risk of hematoma.

Postoperative thromboprophylaxis is the step of perioperative management which received the greatest focus in fast-track surgery [129]. For practical reasons, we report findings about it in separate tables.

A prospective clinical study of 632 primary TJA patients evaluated the best combination between mechanical treatment (compression) and pharmacological chemoprophylaxis (aspirin and/or low molecular weight heparin) to prevent postoperative thromboembolic events (Table 5) [193].

**Table 5:** Mechanical and chemical thromboprophylaxis in 632 primary TJA patients

Is the incidence of readmissions resulting from VTE and bleeding complications higher with LMWH or with mobile compression plus ASA?	In THA, there was no difference in the frequency or readmissions for bleeding complication (wound or systemic) between the treatments. In TKA, patients treated with LMWH had higher readmission rates within 6 weeks after surgery, because of bleeding complications, wound infection, or VTE.
Is the incidence of wound bleeding complications higher with LMWHG or with mobile compression plus ASA?	In THA, there was higher wound bleeding complication frequency with LMWH. In TKA, patients treated with LMWH had a higher frequency of wound bleeding complications or infection.
In TKA, is the frequency of systemic bleeding events and complications related to chemoprophylaxis higher with LMWH or with mechanical compression plus ASA?	Patients treated with LMWH had higher rates of systemic bleeding or complications.
Is there a difference in symptomatic VTEs between LMWH and mechanical compression plus ASA?	There was no difference between the rate of symptomatic VTEs between the groups.

The evaluation was performed answering four questions.

According to these results, the authors advocate routine use of mobile mechanical compression devices, which are able to prevent those VTEs and complications associated with chemical anticoagulants. This is consistent (i) with recommendations of the American Academy of Orthopedic Surgeons and the American College of Chest Physicians [194, 195], (ii) with a retrospective study of 3379 patients which confirmed mechanical treatment with IPCD to be a fast, easy, cheap, and safe support to postoperative multimodal approach [196], and (iii) with clinical pillars for TJA in a bundled payment paradigm [12].

With regard to accelerated pathways, the European Guidelines on VTE prevention were formulated cross-referencing the risk of the patient with the risk of the procedure, adopting Caprini score as an assessment criterion [22, 197] (*).

We summarize them in the following table (Table 6).

**Table 6:** VTE prevention in fast-track and day-surgery orthopedic procedures

	Low VTE risk procedure	High VTE risk procedure
Low VTE risk patient	GT PT only in case they have to rest in bed for more than two days, due to particular reasons	GT + PT (LMWH/AAS) and/or MT
High VTE risk patient	GT + PT (LMWH/AAS) and/or MT	GT + PT (LMWH) + MT

General thromboprophylaxis (GT), early ambulation, optimal hydration, pharmacological thromboprophylaxis (PT), low molecular weight heparin (LMWH) and/or aspirin (AAS), mechanical thromboprophylaxis (MT), intermittent pneumatic compression for high-risk of bleeding

The benefits of early mobilization and the combination between mechanical measures and aspirin are confirmed by other studies, both in fast-track [198] (*) and in conventional pathways [199].

The same guidelines underline the option, in selected procedures, to limit thromboprophylaxis treatment only to the period of hospitalization, and suggest to extend the treatment to up to 4 weeks in high-risk patients and procedures.

The first recommendation is based on a weak level of evidence (2C), but is supported by a large prospective study on 17,582 fast-track TJA procedures according to which 90-day incidence of VTE after in-hospital treatment was only 0.40% [53] (*). On the contrary, the second recommendation is based on a moderate level of evidence (2B), and supports the hypothesis of the prospective study: extended treatment for LOS more than 5 days and high-risk patients is strongly recommended.

Other studies suggest warfarin-based chemoprophylaxis for 6 weeks after THA [200], as well as fish oil as a cost-effective supplement to aspirin after TKA [201], prompting for prospective studies to confirm these suggestions.

## Discussion and limitations

The purpose of this investigation was to identify information to support blood management strategies in fast-track TJA pathways. The investigation confirmed the lack of comprehensive reviews on the topic, which we divided according to recommendations in preoperative, intraoperative, and postoperative.

The need for blood transfusions remains the most significant predictor of a longer stay (more than 3 days) also within accelerated pathways, occurring between 12% and 22% of THA and TKA. However, it seems not to compromise a high patient satisfaction, a fundamental assessment of quality in fast-track procedures. Waiting for blood transfusions is indeed one of the main reasons for a delay in discharge.

Identifying the reasons behind it makes it possible to assess the best clinical and logistical solution for the patient, starting preoperatively. The need for transfusion is associated with age, comorbidities, ASA score, and anemia (with iron deficiency as the most common and reversible cause). In general, a classic transfusion trigger had been established when postoperative hematocrit level at the first day is 25% less than preoperative, while more recent studies found preoperative iron supplement to be a cost-effective solution for non-anemic THA patients. However, fast-track procedures are becoming more and more inclusive, provided that a multidisciplinary management of risk factors is implemented. TKA patients aged ≥ 65 show a weak but significant association between postoperative Hb and 6-min walk test early after surgery, but not with other functional criteria nor quality of life. Postoperative anemia, blood transfusions, and lack of mobilization are the main medical causes of delayed discharge (27.3 more than 4 days) for THA patients aged ≥ 85, while preoperative anemia is the main medical cause of readmission of the same population within 90 days after the intervention, together with suspected (but disproved) thromboembolic events. Finally, stroke is a recognised event which can occur after both TKA and THA procedures: it is therefore fundamental to check for preoperative anticoagulants to avoid cardiovascular perioperative complications in elderly patients. If these precautions prove successful, fast-track pathways realize the possibility of including older patients in major joint replacement programs [202].

Intraoperatively, tranexamic acid is probably the simplest, safe and cost-effective innovation also in case of fast-track procedures, for it almost eliminated the need for other blood conservation strategies. Its benefits increase when combined (i) with local infiltration analgesia, both in hip and knee replacement, as it produces a significant reduction in postoperative Hb levels, pain and length of stay, together with better rehabilitation outcomes; (ii) with low-dose epinephrine in THA, producing a reduction of 180 mL blood loss within 24 h after surgery. The benefits of minimally invasive surgery on blood loss are still debated, and more studies are needed to evaluate its impact on patient satisfaction in fast-track TJA. With regard to TKA, the use of a tourniquet reduces bleeding during surgery, but produces no benefit on knee extension, postoperative Hb levels, pain, and nausea; studies are evaluating its use in combination with multimodal anaesthetic and analgesic regimens. General anesthesia is equivalent to spinal anesthesia with regard to blood loss, but the former dominates the second on several other outcomes, including early mobilization, amount of opioid consumption, and pain scores 6 h after surgery.

Postoperative thromboprophylaxis is the step which has received greater focus. The European Guidelines on VTE prevention suggest different combinations according to patient characteristics and procedures, underlining the benefits of early mobilization together with mobile mechanical compression and pharmacological treatment. Traditional icing provides a high cost-effective benefit on several postoperative outcomes, such as pain, edema, and blood loss, reserving more complicate cryotherapy devices only to selected patients. Finally, removal of drains is safe and feasible within 6–12 h after TKA, although clinical experts question their usefulness.

Several strategies and treatments such as hypotensive epidural anesthesia, topical fibrin sealants, platelet-rich plasma, sealing femoral tunnel, and limb position shows no variation between conventional or fast-track procedures.

A methodological limitation of the study is its partial reproducibility, as the fast-track information achieved from the first electronic search is integrated with information from further searches, previous knowledge, article references, or clinical tips. This is also why the study may not be exhaustive. However, this methodological limitation likely compensates for the lack of information and systematic approach on several topics in this field.

## Conclusion

Many blood management strategies have been put forward to support safe and cheaper orthopedic elective TJA procedures. This article provides a roadmap to more uniform blood management in fast-track orthopedic pathways, pointing out current evidence as well as suggestions for further research.

The difference between conventional and fast-track pathways does not lie in the issues of blood management, which are related to surgical procedures, materials and patients, but in the way in which these contents are integrated with each other, since elective orthopedic procedures offer significant room for planning.

The more fast-track clinical studies are able to add evidence, the greater the benefits for patients, providers, and funders. The next step, therefore, is to put theory into practice [203].

## Data Availability

Not applicable.
